# The LIFE child study: a life course approach to disease and health

**DOI:** 10.1186/1471-2458-12-1021

**Published:** 2012-11-22

**Authors:** Mirja Quante, Mara Hesse, Mirko Döhnert, Michael Fuchs, Christian Hirsch, Elena Sergeyev, Nora Casprzig, Mandy Geserick, Stephanie Naumann, Christiane Koch, Matthew A Sabin, Andreas Hiemisch, Antje Körner, Wieland Kiess

**Affiliations:** 1 LIFE Leipzig Research Centre for Civilization Diseases, University of Leipzig, Philipp-Rosenthalstrasse 27, D-04103 Leipzig, Germany; 2 Department of Women and Child Health, Hospital for Children and Adolescents and Centre for Paediatric Research (CPL), University of Leipzig, Liebigstrasse 20a, D-04103 Leipzig, Germany; 3 IFB Integrated Research and Treatment Centre Adiposity Diseases, University of Leipzig, Philipp-Rosenthalstrasse 27, D-04103 Leipzig, Germany; 4 Department of Women and Child Health, Hospital for Child and Adolescent Psychiatry, University of Leipzig, Liebigstrasse 20a, D-04103 Leipzig, Germany; 5 Section of Phoniatrics and Audiology of the Department of Otorhinolaryngology, University of Leipzig, Liebigstrasse 10-14, D-04103 Leipzig, Germany; 6 Department of Paediatric Dentistry, University of Leipzig, Nürnbergerstr. 57, D-04103 Leipzig, Germany; 7 Murdoch Children’s Research Institute and Royal Children’s Hospital, Flemington Road, Parkville, Melbourne, Victoria 3052, Australia; 8 University of Melbourne, Melbourne, Melbourne, Victoria 3010, Australia

**Keywords:** Cohort study, Children, Pregnancy, Life-course, Epidemiology

## Abstract

**Background:**

Profound knowledge about child growth, development, health, and disease in contemporary children and adolescents is still rare. Epidemiological studies together with new powerful research technologies present exciting opportunities to the elucidation of risk factor-outcome associations with potentially major consequences for prevention, diagnosis and treatment.

**Aim:**

To conduct a unique prospective longitudinal cohort study in order to assess how environmental, metabolic and genetic factors affect growth, development and health from fetal life to adulthood.

**Methods:**

The ‘Leipzig Research Centre for Civilization Diseases (LIFE) Child Study’ focuses on two main research objectives: (1) monitoring of normal growth, development and health; (2) non-communicable diseases such as childhood obesity and its co-morbidities, atopy and mental health problems. Detailed assessments will be conducted alongside long-term storage of biological samples in 2,000 pregnant women and more than 10,000 children and their families.

**Results:**

Close coordination and engagement of a multidisciplinary team in the LIFE Child study successfully established procedures and systems for balancing many competing study and ethical needs. Full participant recruitment and complete data collection started in July 2011. Early data indicate a high acceptance rate of the study program, successful recruitment strategies and the establishment of a representative cohort for the population of Leipzig. A series of subprojects are ongoing, and analyses and publications are on their way.

**Discussion:**

This paper addresses key elements in the design and implementation of the new prospective longitudinal cohort study LIFE Child. Given the recognized need for long-term data on adverse effects on health and protective factors, our study data collection should provide magnificent opportunities to examine complex interactions that govern the emergence of non-communicable diseases.

## Background

Population-based cohort studies of children and birth cohort studies have made landmark contributions in identifying genetic and environmental factors that influence health and disease. In recent years, a variety of them have been established worldwide, and more are in progress. Important examples in Europe are the Avon Longitudinal Study of Parents and Children (ALSPAC), the Generations R Study, the Danish National Birth Cohort (DNBC) and the German Health Interview and Examination Survey for Children and Adolescents (KiGGS) [1-4].

### Why is there still relevance for a new population-based cohort study?

The pace of development and change has accelerated exponentially since the beginning of the 20^th^ century and continues today, affecting many aspects of daily modern life. This implies that cohort studies will always reflect relatively contemporary exposures and practices. However, many ongoing birth cohorts began recruitment in the last century [1,3,5]. Ethnicity, different political and cultural backgrounds are associated with differences in disease prevalence. For example, there are large differences in prevalence of overweight and obesity among preschoolers across Europe [6]. Rapid development in research technologies and advancements in electronic data processing have increased the scope of what is possible to measure and include in epidemiological investigations. This emphasizes the need for contemporary population-based cohort studies across different geographical regions.

### Scope of research

The ‘Leipzig Research Centre for Civilization Diseases (LIFE) Child Study’ has been designed to understand how and through what mechanisms and mediators (epi)genetic, metabolic and environmental factors influence health and development in children and adolescents in modern society. The study is a prospective, longitudinal population-based cohort study of urban children from fetal life until adulthood. It focuses on two main research objectives: (1) monitoring of normal growth, development and health; (2) non-communicable diseases such as childhood obesity and its co-morbidities, atopy and mental health problems. Under the ‘life course’ conceptual model, risk factors during pregnancy and childhood alter the trajectories set by these early signals thus raising or lowering the risk of diseases. Therefore, ensuring salutogenic influences could entrain healthful trajectories of energy balance for life [7].

## Methods

### Study area, collaboration and overview

The LIFE Child study is a prospective longitudinal population-based cohort study with a life course approach to health and disease. A multi-professional team, including paeditricians, psychologists, sociologists, economists, psychiatrists, nutritional and sport scientists, ophthalmologists and dentists, has developed the conceptual framework of the study and established an administrative structure for the management and implementation of the study. Extensive assessments are carried out in pregnant women and their offspring and children and their families. The study collects detailed information from clinical examinations, questionnaires and interviews and includes a collection of several types of biological materials at various time points (see Tables 1, 2, 3, 4 and 5). Prenatal examinations are conducted during the 24^th^ to 26^th^ and 34^th^ to 36^th^ week of gestation. Birth is a crucial part of the study to collect cord blood, placental tissue and to obtain information about the labour and delivery from medical records. During the first year of life, data is collected from children at 3, 6 and 12 month of age. Moreover, a representative sample of children and adolescents (up to age 17.99 years) will be approached together with their families. Participants will be followed annually over a period of ten years irrespective of the age at the first participation in the study. In addition, the LIFE Child study focuses in certain disease cohorts and disease free controls in more detail. The aim of these subgroups is to examine aetiological associations with more in-depth methods that cannot be applied in the whole cohort due to time, financial and logistical constraints. So far, there are three disease cohorts: LIFE Child OBESITY, LIFE Child DEPRESSION and LIFE Child ATOPY. In the future, during the course of the study, additional disease cohorts might be conducted.

**Table 1:** Assessments in the LIFE Child HEALTH and OBESITY cohorts

	**LIFE Child HEALTH cohort**			**LIFE Child OBESITY cohort**
**Participant age**	**0-2 years**	**2-6 years**	**≥ 6 years**	**≥ 6 years**
**Anthropometry**	· Weight	· Weight	· Weight	· Weight
	· Height	· Height and sitting height	· Height and sitting height	· Height and sitting height
	· Head, arm, leg, waist, neck, hip, thoracal and abdominal circumference	· Head, arm, leg, waist, neck and hip circumference	· Head, arm, leg, waist, neck and hip circumference	· Head, arm, leg, waist, neck and hip circumference
	· Biparietal diameter	· Skinfolds	· Skinfolds	· Skinfolds
	· Blood pressure	· Blood pressure	· Blood pressure	· Blood pressure
**Clinical exam**	· Clinical history (perinatal and past medical history, medications, immunisations, developmental history)	· Clinical history (perinatal and past medical history, medications, immunisations, developmental history)	· Clinical history (perinatal and past medical history, medications, immunisations, developmental history)	· Clinical history (perinatal and past medical history, medications, immunisations, developmental history, specific details relating to weight)
	· Clinical examination including neurological examination and pubertal assessment	· Clinical examination including neurological examination and pubertal assessment	· Clinical examination including neurological examination and pubertal assessment	· Clinical examination including neurological examination and pubertal assessment
	· Dental examination including dentition and early childhood caries	· Dental examination including dentition and early childhood caries	· Dental examination (jaw models, dental caries, periodontal disease, orofacial pain, bruxism and tongue function)	· Dental examination (jaw models, dental caries, periodontal disease, orofacial pain, bruxism and tongue function)
**Biological samples**	· Blood, urine and hair	· Blood, urine and hair	· Blood, urine and hair	· Oral glucose tolerance test
				· Blood, urine and hair
**Questionnaires**	· See Table 4	· See Table 4	· See Table 4	· See Table 4
**Motor and cognitive development**	· Bayley scales	· Bayley scales (< 3.6 years)	· Motor skills test	· Motor skills test
		· Motor skills test (> 3.6 years)		
**Imaging**	· Cranial ultrasound (3rd month of life)		· 3D-Bodyscan^1^	· 3D-Bodyscan^1^
			· Sonography (heart, intima media thickness, thyroid and kidney^2^, for a sub-sample)	· Sonography (heart, intima media thickness, thyroid and kidney^2^)
				· Magnetic resonance imaging (for a sub-sample^2^)
**Cardiovascular and pulmonary function tests**			Spirometry	· Spiroergometry
				· Spirometry
				· Endopat (for a sub-sample)
**Additional assessments**			· Voice examination^1^	· Accelerometry
			· Prick test^1, 2 ^(LIFE Child ATOPY cohort)	· Resting electrocardiography
			Optical coherence tomography and ophthalmoscopy^2 ^(for a sub-sample)	· Indirect calorimetry (for a sub-sample)
				· Voice examination
				· Electrical bioimpedance analysis
				· Polysomnography (for a sub-sample)
				· Eating behaviour test^2^
				· Optical coherence tomography and ophthalmoscopy^2^
				Prick test^1, 2 ^(LIFE Child ATOPY cohort)

^1^ The assessment is combined with a specific questionnaire. ^2^Planned.

**Table 2:** Assessments in the parents of the LIFE Child participants

**Questionnaires and interview**	**Other assessments:**
· Clinical history	Biological samples:
· Questionnaires: see Table 4	· Blood, urine and hair
	Anthropometry:
	· Weight
	· Height
	· Blood pressure
	Imaging:
	· 3D-Bodyscan
	Additional assessments:
	· Voice examination

**Table 3:** Assessments in the mothers of the LIFE Child BIRTH cohort

	**24**^**th **^**– 26**^**th **^**week of gestation**	**34**^**th **^**- 36**^**th **^**week of gestation**	**Delivery**	**3**^**rd**^**, 6**^**th **^**and 12**^**th **^**month postpartum**
**Anthropometry**	· Weight	· Blood pressure		· Weight
				· Height and sitting height
	· Height and sitting height			
				· Head, arm, leg, waist, neck and hip circumference
	· Head, arm, leg, waist, neck and hip circumference			
				· Blood pressure
	· Blood pressure			
**Imaging**	· 3D-Bodyscan	· 3D-Bodyscan		· 3D-Bodyscan
	· Ultrasound data of fetal size	· Ultrasound data of fetal size		
**Biological samples**	· Blood, urine and hair	· Blood, urine and hair	· Cord blood, biopsies of the placenta and the umbilical cord	· Blood, urine and hair
				· Breast milk
	· Oral glucose tolerance test			
**Clinical exam**	· Family history and history of pregnancy	· Follow-up history of pregnancy		· Follow-up history of pregnancy
				· History of delivery
	· Current medications	· Current medications		· Current medications
**Questionnaires**	· See Table 4	· See Table 4		· See Table 4
				**Assessments in the child (see Table**1**)**

Evaluations during pregnancy are planned in the 24^th ^- 26^th ^week of gestation and in the 34^th ^- 36^th ^week of gestation. During the first year of their offspring’s life three visits in the study centre are planned.

**Table 4:** Content of questionnaires, interviews and psychological tests

	**LIFE Child HEALTH cohort**		**LIFE Child OBESITY cohort**		**LIFE Child DEPRESSION cohort**		**LIFE Child BIRTH cohort**
	**Child participant information **(to be completed by the parent and/or child)	**Parent participant information **(to be completed by the parent)	**Child participant information **(to be completed by the parent and/or child)	**Parent participant information **(to be completed by the parent)	**Child participant information **(to be completed by the parent and/or child)	**Parent participant information **(to be completed by the parent)	**Pregnant woman participant information **(to be completed by the pregnant woman)
**Environment**	Sociodemography^1^		Sociodemography^1^		Sociodemography^1^	Characteristics of family environment^1^	Sociodemography^1^
	Lifestyle (sexual development, addictive behaviour, media use, sports, spare time activities, social interaction …)^1^	Lifestyle (sexual development, addictive behaviour, media use, sports, spare time activities, social interaction …)^1^	Lifestyle (sexual development, addictive behaviour, media use, sports, spare time activities, social interaction …)^1^	Lifestyle (sexual development, addictive behaviour, media use, sports, spare time activities, social interaction …)^1^	Lifestyle (sexual development, addictive behaviour, media use, sports, spare time activities, social interaction …)^1^		Lifestyle (sexual development, addictive behaviour, media use, sports, spare time activities, social interaction …)^1^
	Quality of life (≥ 10 years)^1^		Quality of life^1^		Quality of life^1^		
			Impact of health conditions^1^				
	Physical and psychological well-being, autonomy and parents, peers and social support and school environment (> 8 years)^1^		Physical and psychological well-being, autonomy and parents, peers and social support and school environment (> 8 years)^1^		Physical and psychological well-being, autonomy and parents, peers and social support and school environment (> 8 years)^1^		
						Parental stress^1^	
					Peer relationship^1^		
					Parenting^1^	Parenting^1^	
**Physical Health**	Health-related symptoms^1^		Health-related symptoms^1^		Health-related symptoms^1^	Physical health Screening^1^	
	Alternative medicine^1^		Alternative medicine^1^				Alternative medicine^1^
	Allergies^1^		Allergies^1^		Allergies^1^		Allergies^1^
	Sleep behaviour^1^	Sleep behaviour^1^	Sleep behaviour^1^	Sleep behaviour^1^	Sleep behaviour^1^	Sleep behaviour^1^	Sleep behaviour^1^
	Child feeding (2–13 years) Eating disorders (≥ 8 years)^1^		Child feeding (2–13 years) Eating disorders (≥ 8 years)^1^				Nutrition diary
			Nutrition diary^1^				
					Pubertal status^1^		
					Stress regulation^3 ^(incl. salivary cortisol samples)		
**Family and School**		Self-assessed partner-attachment^1^		Self-assessed partner-attachment^1^			Self-assessed partner-attachment^1^
	Performance at school (≥ 6 years)^1^		Performance at school^1^		Performance at school^1^		
**Mental Health**					Symptom Screening^1^	Mental health Screening^1^	
			Attention deficit/Hyperactivity symptoms^1^		Attention deficit/Hyperactivity symptoms^1^		
			Perception of teasing (≥ 10 years)^1^				
	Life events (≥ 10 years)^1^		Life events (≥ 10 years)^1^		Life events, neglect, physical/emotional/sexual abuse^1,2^		
	Mental disorders (≥ 18 years)^1^	Mental disorders^1^	Mental disorders (≥ 18 years)^1^	Mental disorders^1^	Psychiatric diagnoses^2^		Mental disorders^1^
	Illness behaviour and somatoform disorders (9 and 12 years)^1^		Illness behaviour and somatoform disorders (9 and 12 years)^1^		Somatic complaints^1^		
					Anxiety symptoms^1^		
					Depressive symptomes^1^		
**Personality**	Personality (Big five concept) (≥ 18 years)^1^	Personality (Big five concept)^1^	Personality (Big five concept) (≥ 18 years)^1^	Personality (Big five concept)^1^			Personality (Big five concept)^1^
	Risk and sensation seeking behaviour (≥ 4 years)^1^		Risk and sensation seeking behaviour^1^				
	Control and competence beliefs (≥ 4 years)^1^		Control and competence beliefs^1^				
	Strengths and weaknesses (≥ 3 years)^1^		Strengths and weaknesses^1^				
	Body attitudes (**≥** 8 years)^1^		Body attitudes (**≥** 8 years)^1^				
					Social competence^1^		
					Self perception^1^		
					Intelligence^3^		

^1^Questionnaire. ^2^Interview. ^3^Test.

**Table 5:** Biobank blood sample collection and parameters of direct analysis

**Participant age**	**Maximum volume of stored aliquots**	**Direct analysis**
**Birth**	24 x 0.3 ml (EDTA, SG)	Not performed
**3 Month**	4 x 0.3 ml (EDTA, SG)	Electrolytes, bilirubin, protein, creatinin kinase, lipid profile, full blood count, thyroid function test, proinflammatoric cytokines, bone parameters, vitamins, NT-pro-BNP, growth hormones
**6 Month**	6 x 0.3 ml (EDTA, SG)	Electrolytes, bilirubin, protein, creatinin kinase, lipid profile, full blood count, thyroid function test, proinflammatoric cytokines, bone parameters, vitamins, allergy testing, troponin T, NT- pro-BNP, growth hormones
**1 – 3 years**	8 x 0.3 ml (EDTA, SG)	Electrolytes, protein, creatinin kinase, lipid profile, full blood count, thyroid function test, proinflammatoric cytokines, bone parameters,vitamins, allergy testing, troponin T, NT- pro-BNP, growth hormones, liver function tests, renal function tests, transferrin, sex steroids
	3 ml (Blood RNA tube)	
**3 – 6 years**	10 x 0.3 ml (EDTA, SG)	Electrolytes, protein, creatinin kinase, lipid profile, full blood count, thyroid function test, proinflammatoric cytokines, bone parameters,vitamins, allergy testing, troponin T, NT- pro-BNP, growth hormones, liver function tests, renal function tests, transferrin, ferritin, sex steroids
	3 ml (Blood RNA tube)	
	4 ml (Cell preparation tube)	
**6 – 8 years**	12 x 0.3 ml (EDTA, SG)	Electrolytes, protein, creatinin kinase, lipid profile, full blood count, thyroid function test, proinflammatoric cytokines, bone parameters,vitamins, allergy testing, troponin T, NT- pro-BNP, growth hormones, liver function tests, renal function tests, transferrin, glucose, insulin, sex steroids
	3 ml (Blood RNA tube)	
	4 ml (Cell preparation tube)	
		**LIFE Child OBESITY cohort and controls :**
		+ Oral glucose tolerance test, proinsulin, c-peptide, uric acid
**8 – 14 years**	20 x 0.3 ml (EDTA, SG)	See 6 – 8 years
	3 ml (Blood RNA tube)	
	8 ml (Cell preparation tube)	
**> 14 years**	26 x 0.3 ml (EDTA, SG)	See 6 – 8 years
	3 ml (Blood RNA tube)	
	8 ml (Cell preparation tube)	
**Parents**	24 x 0.3 ml (EDTA, SG)	Not performed
	3 ml (Blood RNA tube)	
	8 ml (Cell preparation tube)	
**Pregnant Women (24**^**th **^**- 26**^**th **^**week of gestation, 34**^**th **^**- 36**^**th **^**week of gestation and visits postpartum)**	30 x 0.3 ml (EDTA, SG)	Electrolytes, protein, creatinin kinase, lipid profile, full blood count, thyroid function test, proinflammatoric cytokines, bone parameters,vitamins, allergy testing, growth hormones, liver function tests, renal function tests, transferrin,glucose, insulin, sex steroids, oral glucose tolerance test
	3 ml (Blood RNA tube)	
	8 ml (Cell preparation tube)	
		34^th ^-36^th ^week of gestation: no oral glucose tolerance test
		Visits postpartum: no renal function tests

Number of samples x volume collection. *EDTA*, Ethylene-Diamine-Tetra-Acetic acid tube. *SG*, Serum Gel tube. *LH*, Lithium Heparin tube. *NT-pro-BNP*, N-terminal pro-Brain Natriuretic Peptide. *RNA*, Ribonucleic acid.

The LIFE Child study is part of LIFE, a life sciences research program of the University of Leipzig (Leipzig Research Centre for Civilization Diseases - LIFE). The measurements are performed in a well-equipped research centre located on the premises of the University Hospital of Leipzig. Big advantages are the proximity to the Hospital for Children and Adolescents, Department of Women and Child Health of the University of Leipzig, the LIFE Adult cohort and the IFB (Integrated Research and Treatment Centre Adiposity Diseases), as well as the basic research laboratories, hence allowing close interaction and addressing the objectives in an interdisciplinary approach. The LIFE Child study maintains a close collaborative network with the University of Leipzig, the Centre for Paediatric Research (CPL), the IFB, public health centres, schools and kindergartens, the faculty of education, the institute for medical informatics, statistics and epidemiology, the Helmholtz centre for environmental research, the CrescNet® network and with other national and international epidemiological research groups.

### Study cohort

The LIFE Child study will recruit a sample of 10,000 children and adolescents (LIFE Child HEALTH) and their families and 2,000 pregnant women (LIFE Child BIRTH) and their partners between July 2011 and July 2014. All children with their families and pregnant women of the area of Leipzig are eligible to participate in the LIFE Child study. This sample is supposed to represent the population of the city. The LIFE Child study consists of three disease cohorts: The LIFE Child OBESITY cohort is a sample of 1,500 obese children and adolescents that will be assessed and compared to a specific control group (N=1,500) with equally detailed phenotyping including metabolic and cardiovascular measurements. The LIFE Child DEPRESSION cohort is gathering data from a sample of 800 young patients (8–14 years) with psychopathological symptoms and a control sample of healthy children (N=400). Both samples will be assessed by means of a multi-informant, multi-method approach in 2-year follow-ups over 10 years. In the LIFE Child ATOPY cohort extra questionnaires and a prick test with common allergens in addition to the LIFE Child HEALTH program will be performed.

### Ethical issues

The LIFE Child study like any other paediatric study encounters complex ethical issues [8]. These include the collection of data from minors, long-term biobanking of biological samples and subsequent genetic and epigenetic analysis and data privacy. The study was designed in accordance to the declaration of Helsinki [9]. The Ethics Committee of the University of Leipzig was involved in the study outline and approved the study (Reg. No. 264-10-19042010). Fully informed and written consent is obtained for all participants and their parents. Starting at the age of twelve, written consent is also obtained from the children themselves. Participants are asked to re-consent at each study visit during complete course of the study. Consent for long-term use of data is sought. Moreover, participants are informed of their right to withdraw without explanations or adverse consequences at any time. A special two-pass encryption program is used to generate subject identifier codes (SIC) for all participants of the study in order to pseudo-anonymise their data. Both an internal and an independent external ethical advisory board have been established.

### Recruitment and attrition

**Figure 1:**
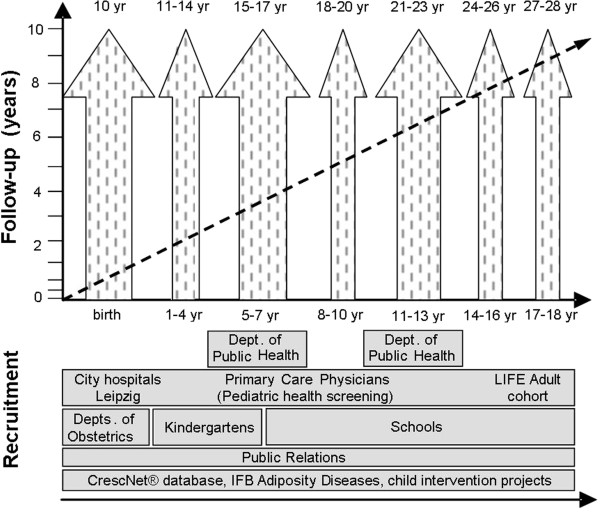
**Strategies of recruitment. **Strategies of recruitment and favoured sampling sizes depending on the age at the first participation.

**Figure 2:**
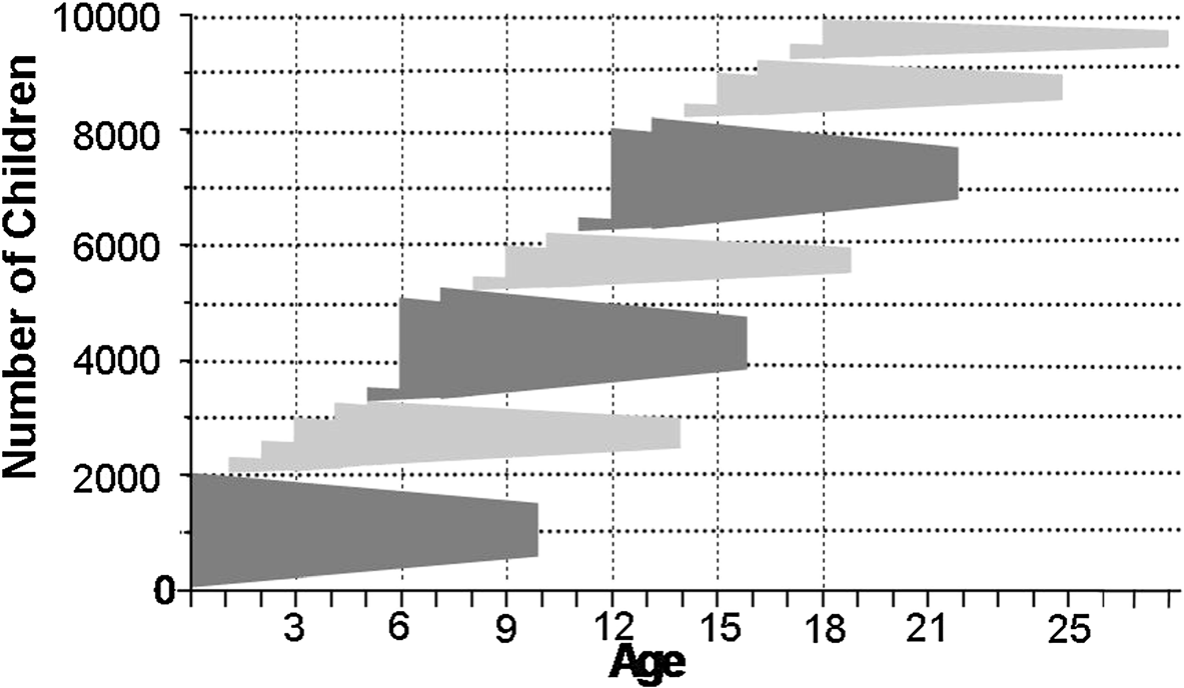
Numbers of participants to be recruited in the different age groups.

**Figure 3:**
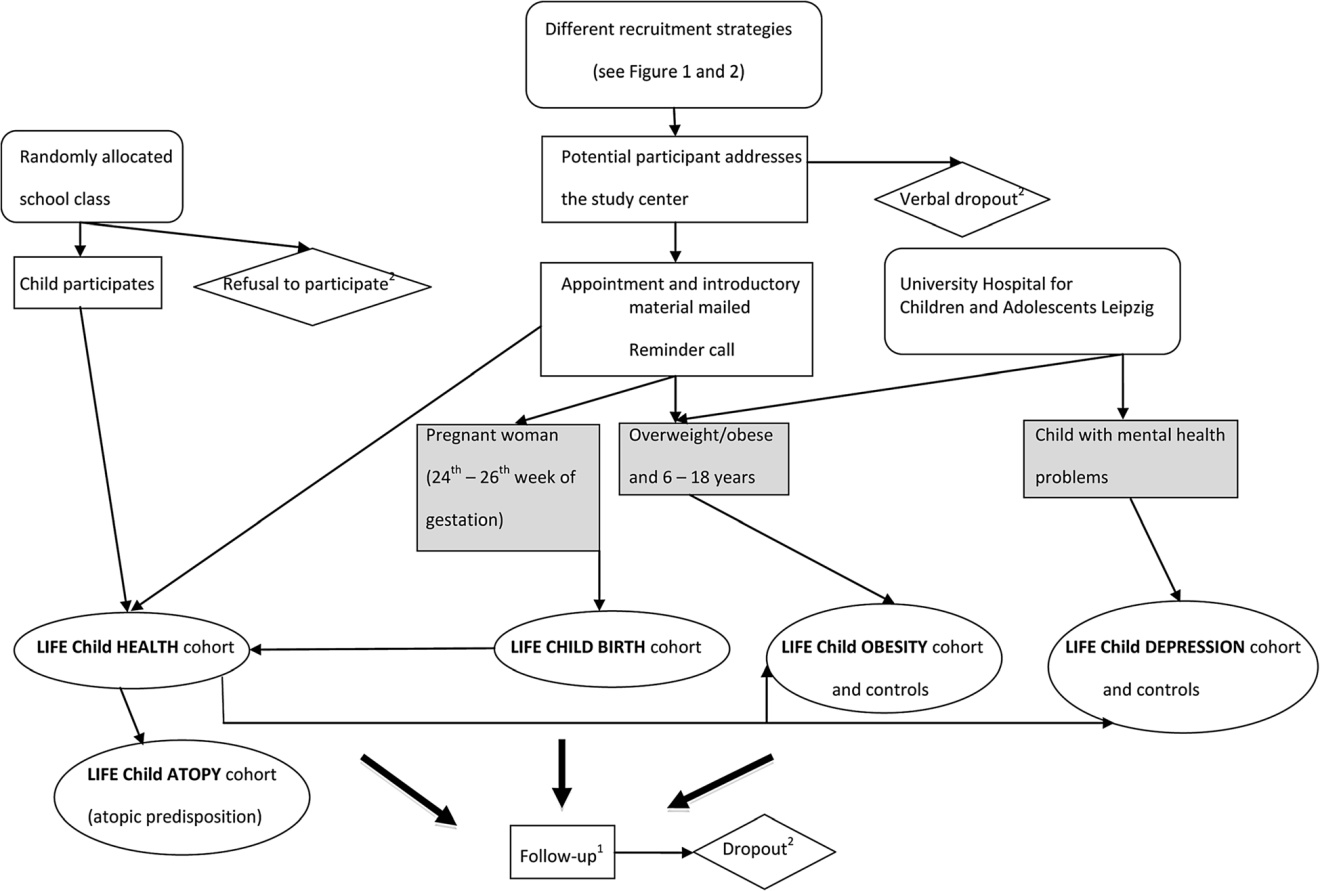
**Recruitment and follow-up procedure.**^1 ^LIFE Child HEALTH cohort and LIFE Child OBESITY cohort: annual follow-up. LIFE Child DEPRESSION cohort: follow-up every two years. LIFE Child BIRTH cohort: follow-up the 34^th^ to 36^th ^week of gestation and the 3^rd ^and 6^th ^month postpartum. ^2 ^Completion of a dropout questionnaire.

**Figure 4:**
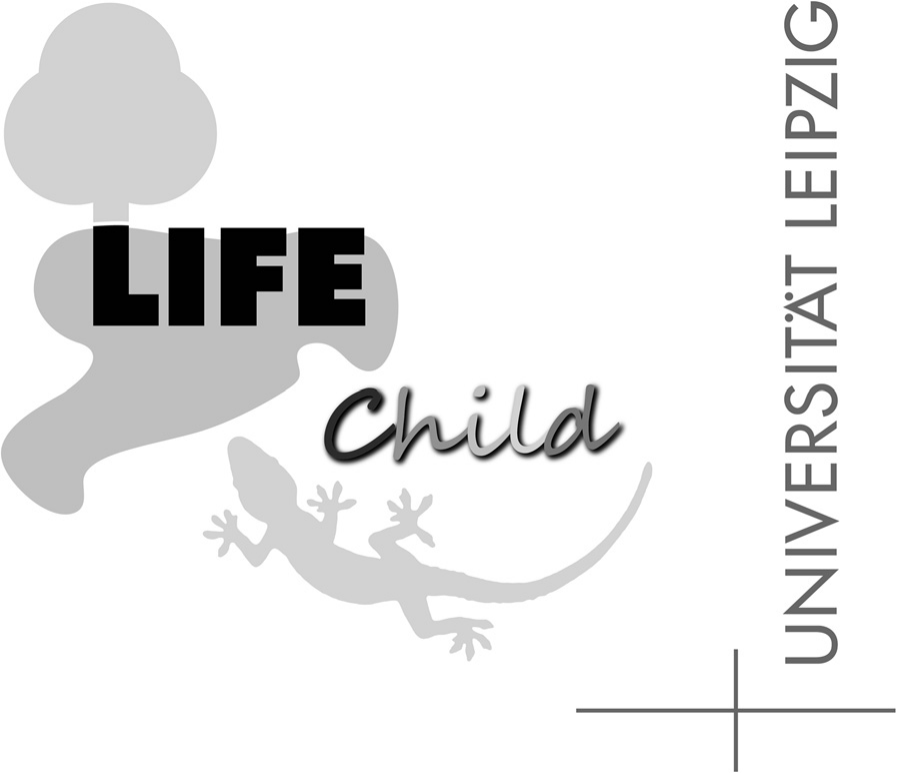
The LIFE Child study logo.

The LIFE Child study decided to employ a variety of recruitment strategies. The overall approach is a community-based collaborative network of university hospitals, local clinics, public health centres, kindergartens, schools and partner study centres like the IFB (see Figures 1, 2 and 3). Specific contact information is given to participants at the time of recruitment. Telephone and email reminders are performed. A variety of items embossed with the study logo (see Figure 4), a green amphibian, as well as small monetary incentives (not more than 20 Euro per child) are given to participants at the time of recruitment to compensate for their time [10]. Pregnant women are also offered an ultrasound video of their baby. Recruitment goals are continuously monitored and strategies are adapted. The researchers send Christmas and birthday cards and newsletters to encourage continued participation. Study promotion like presentations, forums, fliers/posters, press releases and TV spots is targeted at potential participants. Regular feedback on results will be provided. As retention involves building relationships with participants several events like children’s parties and sport events (e.g. running) are arranged. Several German cohort studies have already shown high enrolment and follow-up rates. This might be due to the fact that the German population, especially in East Germany, is apparently more compliant with the participation in research projects in comparison with other countries [5,11]. People who voluntarily contact the study centre for participation tend to be different from other people in terms of basic demographic factors (e.g. social class, level of education and core personality traits). To minimize this bias, whole school classes, especially from the inner cities with a lower social background are approached. Dropouts and refusals to participate can lead to selection bias due to differences between participants who remain in the study and dropouts [12]. Therefore, questionnaires requesting the underlying causes will be sent to the families and several research projects addressing dropout issues have been initiated.

### Assessments

There is a basic program that will be carried out at each study centre visit. This program includes clinical history (perinatal and past medical history, medications, allergies, immunizations, developmental history and family history) clinical examination, collection of blood, hair and urine samples, anthropometry and different age-dependent questionnaires (see Table 4). Questionnaires are completed by the parents and starting at the age of eight years also by the child itself. They are standardized, validated, published, computerized and piloted. Whenever possible mother, father and even the teacher of the child are asked to answer the questionnaires separately. Additional assessments are added at different ages or in subgroups. The development of children beginning at the age of three months until 3 ½ years of age is evaluated by psychologists using the newly developed third edition of the Bayley Scales of Infant and Toddler Development® (Bayley-III®) [13]. In close collaboration with the developers of these scales – Pearson Clinical Assessments – and other study centres, LIFE Child study will contribute to the German standardization of this new edition. Beginning at the age of 3 ½ years, all children attend a motor skills examination. At the age of six years, lung function testing (spirometry), a voice examination and a body scan are added. The 3D body scanner (VITUS XXL 3D) is a measuring tool for a rapid quantification of body shape [14]. In LIFE Child study, it is used for anthropometric investigations. Within a few seconds, the whole body of the participant is scanned with non-hazardous laser beams and a 3D figure appears on the attached computer screen. The results of the body scanner will be compared to the results of the classic anthropometry to evaluate whether the body scanner can replace measuring tape, scales and stadiometers in the future. Another unique assessment is the voice examination, which will allow to explore genetic and environmental effects on the voice [15]. Voice limits are measured in a standardized fashion (sound-pressure levels in dependence of frequency) when the participants speak and sing. These data will provide clinically relevant information about the volume and pitch range of the voice [16]. The often underestimated oral diseases have profound effects on general health and quality of life [17]. The social impact of diseases like dental caries, periodontal diseases and orofacial pain in children is substantial. Worldwide more than 50 million school hours are lost each year to dental-related illness and especially children with lower social background are affected [18]. Therefore, dental examinations including jaw models and evaluation of dental caries, periodontal disease, orofacial pain, bruxism and tongue function are performed. In the LIFE Child OBESITY cohort (including the control group), additional parameters such as electrical bioimpedance, assessment of basal metabolic rate, oral glucose tolerance test, spiroergometry and measurement of endothelial function using EndoPAT® are assessed. The oral glucose tolerance test (oGTT) is performed with insulin and glucose measured every 15–30 min between 0 and 120 min. It is assumed that this approach with close measurement intervals will allow a better insight into metabolic processes. The EndoPAT® 2000 (Itamar Medical Inc., Caesarea, Israel) is a device to measure peripheral arterial tonometry – PAT – signals. It has been validated and used in several studies to analyze the reactive hyperaemia index (RHI), which quantifies the dilatation response of peripheral vessels to increased blood flow following suprasystolic occlusion [19-21]*.* A low RHI has been shown to be associated with endothelial dysfunction and a higher risk for existent or future cardiovascular events [22]. Tables 1, 2, 3, 4 and 5 show all the parameters collected in the LIFE Child study. More detailed information on the LIFE Child study protocol is available upon request and on our homepage (http://www.uni-leipzig-life.de) to any researcher interested in the program.

### Biological samples

In accordance with the COBRA project (Childhood Overweight BioRepository of Australia project) the LIFE Child study team established a unique ‘biorepository’ of data and biological samples of the cohort [23]. Blood, urine and hair samples are collected from each study subject and its parents annually. The volume of the blood withdrawal has been adjusted to the age and body weight of the children. Some blood parameters are analyzed directly. These parameters cover several areas of particular health interest like micronutrient deficiency, sero-epidemiology, growth and sexual hormones and risk indicators for different diseases (see Table 5). To minimize the possibility of degradation, the plasma and serum samples for future investigations are biobanked in small aliquots (300 μl) and stored at −140°C [24]. Genomic DNA, dried blood spots, paxgenes (for RNA isolation and expression/eQTL studies) and immortalized lymphocytes are stored at −80°C. At delivery umbilical cord blood, the umbilical cord itself and placental biopsies are stored in the biobank. The biopsies are stored at −80°C, while the cord blood is aliquoted and biobanked at −140°C. Breast milk is known to contain many bioactive hormones and peptides, which may play important roles in neonatal health and development. For example researchers of the LIFE Child study team could show that infants’ body weight gain may be influenced by milk leptin concentrations [25]. Breast milk is collected and stored at −80°C from lactating mothers the third and sixth month postpartum.

### Quality management issues

For epidemiological research, accuracy and reliability of the measurements used are extremely important considerations. Quality management in the LIFE Child study involves multiple levels of training, monitoring and feedback activities, including: certification of research personnel for all specialized testing procedures and for data entry, documentation of all procedures in a written and web-accessible manual of procedures and ongoing monitoring of data quality. Several feasibility studies have been carried out to evaluate different assessments. A pilot study was performed to test the complete study protocol. This project with 200 normal weight and 200 obese participants was finished in December 2011. The experiences have been applied to optimize the operational sequences of the LIFE Child study. Since the quality of data from participants of the pilot phase was comparable to those collected later on, data have now been included in the total cohort. All different assessments are performed and evaluated by a well-trained team of physicians, nurses, technical assistants, psychologists, dentists, sport and nutritional scientists. Documents outlining the standard operating procedures (SOPs) exist for all assessments. The completeness, consistency and plausibility of all collected data are controlled regularly and in a standardized fashion using computation of the data. In addition to internal quality management, an external scientific advisory board was established to supervise the study.

### Incidental findings and disclosure of results

Newest research technologies applied in large cohorts may lead to unexpected results. These incidental findings are defined as unexpected findings discovered in the course of research but ‘beyond the aims of the study’ [26]. Policies addressing various categories of findings and plans for their handling have been established. At the end of each study visit the participating families are invited to a personal conversation with one of the physicians to receive a short feedback on the results of the routine measurements, such as physical measures (weight and height). Any results that meet the requirements of scientific validity, clinical significance and benefit, such as prevention or treatment measures are reported to the family and with the parent’s consent also to the family’s primary care physician [27,28]. Additional diagnostic or therapeutic steps are given to the discretion of the family and fall into the responsibility of the family’s primary care physician. In cases of doubt whether a finding should be reported to the families or not, the ethical advisory boards and medical experts are being asked for advice and their decisions are documented for future decision taking. When situations of suspected child abuse/neglect are encountered the LIFE Child team is aware of legal obligations to report this information to authorities.

### Research projects

A short description of the specific aims, measurements and projects of the LIFE Child study is given here:

1. Growth, development and health (the LIFE Child HEALTH cohort)

Genetics and perinatal conditions such as social and environmental determinants are major factors influencing growth, development, health and disease outcomes including all-cause mortality and cardiovascular disease [29]. Some of the research questions that will be addressed are:

(1) Which fetal and infant growth patterns are associated with the development of risk factors for non-communicable diseases such as obesity, cardiovascular diseases and atopy?

(2) Which environmental factors and genetic variants are determinants of normal and abnormal growth, development and health?

(3) How are normal growth, physical activity and cognitive development defined in modern society?

Measurements planned and biological samples collected in the LIFE Child HEALTH cohort are listed in Table 1,4 and 5.

2. Pregnancy and offspring (the LIFE Child BIRTH cohort)

The LIFE Child study also includes a birth cohort. In this cohort the influence of antenatal factors on growth and neurobehavioral outcomes will be investigated. Prenatal visits are conducted during the 24^th^ and the 26^th^ week of gestation with a follow up ten weeks later (see Table 3). During the first year of life, data from newborns is collected at 3, 6 and 12 month of age. The child is then integrated into the LIFE Child HEALTH cohort. In order to enable and improve the collaboration with other birth cohorts, the LIFE Child BIRTH cohort is already listed on the website birthcohorts.net [30]. Research questions that will be addressed include:

(1) What are the interactions between gestational environmental factors (e.g. maternal diet) and susceptibility genes in the development of non-communicable diseases such as obesity, cardiovascular diseases and atopy?

(2) What specific determinants underlie the differences in birth outcomes?

(3) What are the prenatal and early postnatal determinants of normal and abnormal growth, physical and cognitive development?___

3. Childhood overweight and obesity (the LIFE Child OBESITY cohort)

The rates of overweight and obesity are unacceptably high, with significant sequelae for the health and well-being of affected children. The pathogenesis of obesity related comorbidities such as metabolic disorders and cardiovascular disease already starts in childhood [31]. Therefore, identifying predisposing and protective environmental and genetic factors and subsequent prevention and therapy of early onset obesity have become a major focus in research [32,33]. The LIFE Child OBESITY cohort will be established as a well-characterized cohort of overweight and obese children and adolescents compared to healthy normal weight controls (see Table 1). All participants also undergo the complete LIFE Child HEALTH program. For the purposes of recruitment overweight and obesity are classified using the 90^th^ and 97^th^ body mass index (BMI) percentile cut-offs of Kromeyer-Hauschild, as these classification is routinely used in Germany [34,35]. Worldwide several other systems for classification, such as the World Health Organization (WHO) criteria or the ones of the International Obesity Task Force (IOTF) exist [36]. Therefore, the LIFE Child study always includes a data set with raw height, weight and BMI to enable researchers to use other classifications when it comes to data analysis. Some of the research questions that will be addressed are:

(1) What are the biological, genetic, social and behavioural determinants and consequences of childhood obesity?

(2) Which specific factors lead to an increased risk of later weight-related comorbidities?

(3) Which genetic and environmental determinants are associated with long-term weight loss or weight maintenance?___

4. Mental health (the LIFE Child DEPRESSION cohort)

Depressive disorders are more and more present even in young children. The most prevalent problems are behavioural and emotional disorders and hyperactivity [37]. Since nowadays health sciences focus on a salutogenetic approach, the quest for protective factors on mental development and health has increasingly gained importance [38]. Several genetic, demographic, psychosocial, cognitive and biological correlates of onset and course of mental health problems such as early-onset depression have already been identified. Unfortunately, little is known about the interactions of these factors [39]. Research questions that will be addressed include:

(1) What are determinants of normal and abnormal mental and social development?

(2) What are determinants of depressive disorders in children, adolescents and young adults?

(3) What influences the developmental course of emotional symptoms and affective disorders between childhood and early adulthood?

Measurements planned in the LIFE Child DEPRESSION cohort are listed in Table 4.

5. Atopy (the LIFE Child ATOPY cohort)

Asthma and allergies have become increasingly prevalent during recent years [40,41 -42]. The rapid increase of their prevalence in East Germany after the German reunification implies an association with lifestyle changes [43]. Therefore, the family history and questionnaires concerning atopic symptoms together with blood parameters like IgE concentrations specific for different food allergens and aeroallergens are assessed in the LIFE Child HEALTH cohort and allow to define a subcohort of clinically manifested or predisposed participants. In this LIFE Child ATOPY cohort additional questionnaires and a prick test with common allergens will be performed. Research questions include:

(1) What is the overall incidence of atopy from birth to adulthood?

(2) What are the underlying interactions between environmental and genetic determinants that lead to atopy?

### Results

**Figure 5:**
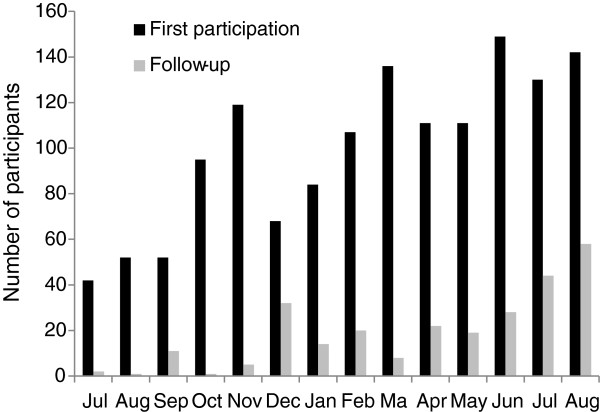
**Steady increase of study participant numbers and follow-up visits over time. **July 2011 to August 2012.

Close coordination and engagement of a multidisciplinary team in LIFE Child successfully established procedures and systems for balancing many competing study and ethical needs. By means of quality management, daily operating procedures have been improved before data collection for the main study of LIFE Child started in July 2011. Until August 31^st^ 2012, 1,382 children and their families and 112 pregnant women have attended at least one appointment in the study centre and 212 follow-up visits have already been executed. Moreover, 96 babies were enrolled at birth. In the LIFE Child DEPRESSION cohort 207 children (including clinic-referred and healthy children) participated. We were literally overwhelmed by the success of our recruitment strategies with 534 families still being on the waiting list for participation in the LIFE Child study. Figure 5 shows the steady increase of participant numbers over time. Initial data suggest that the cohort will be representative for the population of Leipzig and indicate a high acceptance rate of the study program and the different assessments. For example, preliminary data on the acceptance revealed that the overall response was very positive with 89% of the children and their families planning to definitely come again for follow-up. Detailed analyses on dropout rates and the acceptance of the study program are on the way and will be published elsewhere. Initial data from school classes enrolled in the study show that boys are more likely to attend lower level education schools than girls while girls are overrepresented in the higher educational tracts. Data on the overall frequency of incidental findings, the gender- and age-specific distribution and the frequency with which clinical action is taken as a result of discovering an incidental finding are going to be reported elsewhere.

## Discussion

Like any other comparable study, the LIFE Child study has its strengths and limitations. Some of the main strengths are the large sample size of the cohort, the longitudinal design of the study and the broad age range. Longitudinal studies in general have many advantages compared to cross-sectional studies like the reduction of retrospective bias from the participants, the possibility to take biological samples at different time points and the monitoring of changes with age [44]*.* Further advantages of the LIFE Child study are the multidisciplinarity and close collaboration with the University of Leipzig and diverse research centres like the IFB, the Helmholtz centre for environmental research and the LIFE Adult cohort. The LIFE Child study has an open policy in regard to collaboration with other research groups. Compared to other planned or recently started population-based cohort studies, the size of the LIFE Child study is not larger but the measurements are more detailed [2,3]. For example biological samples are obtained from all participants and their parents at each follow-up visit. By this a ‘biorepository’ containing a longitudinal collection of samples starting in the early stages of life and childhood from each participant will be established that provides a unique and powerful opportunity to monitor metabolic and epigenetic changes over time [45]. A huge variety of different assessments are performed. Established assessments are combined with newest technology such as the 3D body scanner. These new methods, the combination of various different assessments from many different disciplines performed annually and the possibility to cover the entire lifespan (together with LIFE Adult) make the LIFE Child study unique. The main potential limitation may lie in the difficulty to maintain a representative cohort especially during follow-up [46]. Due to some of our recruitment strategies, the participants of the LIFE study will likely be characterized by a higher educational and socio-economic status compared to the general population. Whole school classes, especially from inner cities, are approached to minimize this bias. This strategy will help to recruit a sample that adequately represents the population of Leipzig. The idea to invite school classes is a relatively new approach in epidemiologic research. However, researchers of the LIFE Child team already successfully performed a cross-sectional cohort study along with regular public health service examinations in a total of 2,365 healthy schoolchildren [11]. The experiences using this sampling technique in mid-Germany are promising and may help to improve the response rate [47]. The aim of LIFE Child is to improve the knowledge about normal and abnormal child growth, development, health and disease in contemporary children and adolescents and thus help to develop superior strategies in the prevention and treatment of diseases. Consequently, the results may improve the situation for future generations of children, adolescents and adults in Leipzig and beyond. The study will provide core material for many research projects and the value of the collected data in the LIFE Child study will increase over time. Researchers from everywhere are invited to participate in data analysis and sharing. Moreover, it is possible for researchers to use the templates of the LIFE Child study to generate other disease cohorts for which the LIFE Child HEALTH cohort may contribute controls.

## Competing interests

The authors declare that they have no competing interests.

## Authors’ contributions

MQ and MH have written the manuscript. AH, AK and WK conceived and designed the study, and led development of study protocols and procedures to obtain ethics approval. AH, SN and CK are responsible for the day-to-day running of the study. MQ, MH, ES, NC, MG, SN and CK have been involved in the development of the study from conception to current practice. MQ, MH, ES, NC, MG, SN, CK and AH are actively involved in recruitment. MD established the LIFE Child DEPRESSION cohort, MF carried out the voice examination and CH implemented the dental examination. WK and MS supervised and reviewed the manuscript. All authors read and approved the final manuscript.

## Funding

The LIFE child study is funded by means of the European Union, by the European Regional Development Fund (ERDF) and by means of the Free State of Saxony within the framework of the excellence initiative for the period 2009–13. Other official funds from the German Research Foundation (DFG) and the federal ministry of education and research (BMBF) have been obtained for subprojects related to intermediate outcomes. Additional funding is being obtained continuously.

## Pre-publication history

The pre-publication history for this paper can be accessed here:

http://www.biomedcentral.com/1471-2458/12/1021/prepub
